# Construction Notes

**Published:** 1893-02-04

**Authors:** 


### CONSTRUCTION NOTES.

The committee of the Falkirk Hospital have agreed that a hydraulic pump shall be obtained for the hospital. The Sanitary and the Hospitals Committee have urged the necessity for the erection of a temporary small-pox hospital, Mr. Nimmo's estimate for the same, containing two wards and six beds, with an apartment for a nurse, being accepted at £128 8s.

The enlargement of the Walmersley Home in connection with the Northern Counties Hospital for Incurables is now completed. The west wing contains bed-rooms, bath-room, lavatory, and offices for the staff, besides a large day and dining-room combined, an excellent larder, and a store for dry goods. The south wing contains six bright, cheerful rooms, and will afford accommodation for twenty additional patients.

The new Fever Combination Hospital to be erected for the county of Alloa is to be built in the Old English style of architecture, and will consist of an administrative block, two main pavilions, probationary wards, and laundry block, and will give accommodation for 24 beds in the main pavilion and four in the probationary wards. The estimated cost of the new building is £5,000, and it is to be erected on the Parkhead Farm. Mr. John Melvin, Alloa, is the architect.

Considerable alterations are to be effected at the Addenbrooke's Hospital, Cambridge, concurrently with the reconstruction of the drainage system, which work, inclusive of incidental expenses, is estimated at about £3,500. The recommendation of the officers of the weekly board concerning the relaying of the floors in the surgical wards was discussed, but eventually dismissed for the time being on the ground of the cost, which was estimated at £800. The condition of the floors was not unanimously considered bad enough to warrant this outlay, considering that the funds of the hospital were not in a sufficiently prosperous condition to meet so heavy an outlay.

